# Influence of parity on gestational and perinatal parameters of jennies of the Nordestino ecotype

**DOI:** 10.1016/j.vas.2026.100768

**Published:** 2026-07-12

**Authors:** I.M. de Farias, J.M. Santiago, G.G. de Sobral, A.H. da Silva, N.A. Felix, D.S.A. Pinto, E.F. de Melo Júnior, K.M.L. da Silva, G.F. Carneiro, V.N. Maia, A.P.G. Pinto, L.L. da Rocha, I.V.F. Gonzaga, F.M.P. Taran, J.E.C. Lucena

**Affiliations:** a Federal University of Pernambuco Agreste – Garanhuns, PE, Brazil; b Federal Rural University of Pernambuco – Serra Talhada, PE, Brazil; c Federal Rural University of Pernambuco – Recife, PE, Brazil; d Federal University of Recôncavo of Bahia - Cruz das Almas, BA, Brazil; e Federal University of the São Francisco Valley – Petrolina, PE, Brazil

**Keywords:** Donkey, Multiparae, Newborns, Primiparae

## Abstract

This study aimed to expand the knowledge on maternal and perinatal parameters of donkeys of the Nordestino ecotype to support clinical and reproductive management. A sample of 29 jennies and their foals was assigned to two groups (primiparous and multiparous) in a completely randomized design. In the prepartum period, the pH of mammary secretion was monitored daily. During foaling and in the first hours of life of foals, gestational parameters including maternal weight, the length of gestation, of parturition, and of placenta release, placenta weight, and placenta/mother, newborn/mother, and placenta/newborn ratios were assessed. Perinatal parameters such as time for sternal recumbency, standing up, showing suckling reflex, first suckling, first urination, and meconium release were also assessed. In both groups, foaling proximity was characterized by a progressive reduction in the pH of mammary secretion, with parturition on average taking place 39 h after pH reached 6.4. No differences (p > 0.05) in gestational parameters were observed between primiparous and multiparous jennies. However, foals from multiparous mothers had higher weight at parturition (18.92 ± 1.99 kg) when compared with those from primiparous jennies (16.35 ± 2.27 kg) (p = 0.004). The former took less time to stand up (multiparous: 22.90 ± 11.63 min; primiparous: 46.25 ± 29.80 min) and for first suckling (multiparous: 57.00 ± 21.62 min; primiparous: 93.50 ± 28.20 min) (p = 0.01). It is concluded that monitoring mammary secretion pH is an efficient method to help predict foaling and that foals from multiparous jennies exhibit neonatal behaviors earlier.

## Highlights

• Mammary secretion pH decline accurately predicted parturition in Nordestino donkeys.
• Parturition occurred ∼39 h after mammary secretion pH reached 6.4.
• Parity did not affect gestational parameters in Nordestino jennies.
• Foals from multiparous jennies were heavier and showed neonatal behaviors earlier.
• Multiparity favored earlier standing and first suckling in donkey foals.

## 1 Introduction

Unlike other donkey breeds developed in Brazil, which have a well-defined breed standard, the Nordestino donkey was mainly selected based on its capacity to survive and thrive in the Caatinga biome that is characteristic of the Semi-Arid portion in the Northeast region of Brazil. For that reason, many authors classify it as an ecotype and not as a breed, characterizing it as a set of individuals adapted to the environment, but bearing morphological variations such as coat and the shape of the head, chanfrin, and ears. That distinction reflects a predominantly physiological and behavioral standardization that favors rusticity, docility, and sturdiness (Ribeiro et al., 2023).

Although a well-defined productive system does not exist for the Nordestino donkey in Brazil, the growing global interest in milk, meat, and hide from donkeys has expanded the outlook of exploring those animals, which requires greater technical knowledge on the rearing and management of the species (Messias et al., 2021).

Assessing perinatal parameters of mares and jennies is key for the early detection of gestational risks, optimization of assistance in foaling, and improvement of neonatal survival since physiological and clinical alterations in late gestation and during parturition may result in low newborn vitality, thus increasing morbidity and perinatal mortality (Abraham & Bauquier, 2021). Furthermore, colostrum quality and the efficiency of passive immunity transfer also play a relevant role in newborn survival and proper development in the first hours of life (Felix et al., 2022). In this context, measuring indicators such as weight at parturition, time to stand up and suckle, Apgar score, behavioral signs, and blood or biochemical variables allows objectively assessing neonatal vitality and adaptation, which helps identify differences between species and define specific clinical protocols for domestic Equidae animals, including equine foals and hinnies.

The success of any animal productive system requires adopting assertive measures since the initial phases of rearing aiming at the healthy and balanced development of newborns (Felix et al., 2022). Knowing the events that involve gestation, foaling, and the immediate postpartum period is especially relevant since maternal and neonatal conditions in those critical periods play a fundamental role both in newborn survival and development and in the well-being of the mothers.

In this context, the present study aimed to expand the knowledge on maternal and neonatal parameters during foaling and the immediate postpartum period of donkeys of the Nordestino ecotype to support the clinical and reproductive management of those animals. It also seeks to promote practices that favor animal well-being and productive efficiency so as to contribute to more sustainable and effective management.

## 2 Material and methods

### 2.1 Ethics committee

The present study was carried out in accordance with the ethical guidelines for the use of animals in scientific research. The protocol was submitted and approved by the Committee of Ethics in Animal Use (CEUA) of the Federal University of the Agreste of Pernambuco – UFAPE under protocol CEUA/UFAPE 8863,110,621 to ensure the well-being and integrity of the animals used in the research.

### 2.2 Area

The experiment was carried out at the facilities of the Experimental Farm of the Federal Rural University of Pernambuco (Universidade Federal Rural de Pernambuco - UFRPE), located in the municipality of Garanhuns – PE at 8°58′31.28″ S and 36°27′15.00″ W.

### 2.3 Animals

Twenty-nine pregnant Nordestino jennies with age estimated between 4 and 10 years old with mean live weight of 184.7 ± 11.9 kg were used. The newborns of those jennies were also included in the study. All jennies underwent a sanitary protocol that included deworming with ivermectin and praziquantel (Eqvalan Gold®) prior to mating, at 120 and 240 days of gestation, and on the day after foaling. They also received booster vaccine shots against rabies and tetanus. The jennies were weighed monthly using a Prix Rebanho (Toledo®) scale to monitor body weight and nutritional adjustments were made if needed aiming at maintaining body score 4 (Carroll & Huntington, 1988) in the final third of gestation.

### 2.4 Experimental design

The animals were divided into two groups according to parity, comprising a group of nine primiparous jennies (between 4 and 5 years old, mean body weight of 178.98 ± 16.37 kg) and a group of twenty multiparous jennies (between 5 and 10 years old, mean body weight of 187.20 ± 15.04 kg), and the data were analyzed using a completely randomized design.

The number of animals in each group reflected the availability of the herd at the time of the study, a common condition in trials carried out in the field with locally adapted ecotypes.

### 2.5 Reproductive management

In order to achieve a uniform parity period, the jennies had their estruses synchronized with the use of a prostaglandin analog (Lutalyse®) applied in two doses 14 days apart. Follicular dynamics were followed using transrectal ultrasound. Follicle growth was followed until the dominant follicle was detected, with diameter ≥ 35 mm, possibly responsive to the use of human chorionic gonadotropin (Vetcor®), which was used to induce ovulation. The semen of a single male donkey of the Nordestino ecotype was employed, which was diluted with a milk-based extender at a 2:1 ratio (BotuSÊMEN®), while the insemination doses were split for the jennies close to ovulation. However, the first conception rate via artificial insemination was unsatisfactory, with only one jenny becoming pregnant.

In face of that, the reproductive protocol had to be adjusted to use natural mating. In this new protocol, follicle control was maintained until the dominant follicle was detected (≥ 35 mm), when ovulation was induced (Vetcor®) and controlled natural mating was carried out. Pregnancy was confirmed via transrectal ultrasound (DP10 Vet Power Mindray®) 15 days after ovulation by identification of the embryonic vesicle.

Foaling took place between February 29th, 2024 and May 18th, 2024. From the group of primiparous jennies, 3 females and 6 males were born, while 7 females and 13 males were born from the multiparous jennies.

### 2.6 Nutritional management and adaptation

The jennies remained in pens with native pasture throughout the gestational period, with water and mineral lick ad libitum. At 11 months of gestation, the animals were transferred to a maternity pen near the facilities for monitoring of mammary secretion pH.

In that pen, with no plant cover, the jennies were fed Tifton 85 (Cynodon dactylon) hay to meet the daily intake needs of 2.0% live weight based on dry matter (NRC, 2007). They also received supplementation with specific concentrate feed for equines in reproduction, which was split into two daily meals, at 7 a.m. and 3 pm., at 0.35% live weight per meal per animal (Table 1), in addition to water and mineral lick ad libitum.

**Table 1:** Diet composition fed to jennies starting on the 11th month of gestation.

Composition (g/kg DM)	Commercial feed	Tifton 85 hay
Dry matter	870.00	828.79
Crude protein	200.00	73.38
Total carbohydrate	ND*	798.22
Neutral detergent fiber	ND	867.43
Acid detergent fiber	180.00	ND
Fibrous matter	120.00	ND

Mineral lick (Coequi Plus®): commercial mineral supplement for adult equids. Composition: Ca 175 g/200 g; P 60 g; Na 120 g; K 20 g; Mg 13.6 g; S 12 g; Cu 1200 mg; Fe 2000 mg; Zn 2200 mg; Mn 970 mg; I 125 mg; Co 21 mg; Se 10 mg; F 600 mg.

*Not Determined.

### 2.7 Foaling prediction

Foaling was predicted by measuring mammary secretion pH using colorimetric strips with a range between 6.4 and 8.0 (Macherey-Nagel®). Mammary secretion pH was monitored from the 11th month of gestation onwards and was performed daily at 7:30 a.m. When pH dropped below 7.0, the jennies started being monitored in maternity bays throughout the night. They were followed remotely so as to prevent stress and impact on parturition. The time parturition started was recorded when the jennies went into recumbency, with the start of fetal membrane rupture (stage II) (Holyoak & Freeman, 2003), which allowed calculating the time, in hours, since the last pH value recorded.

### 2.8 Gestational and perinatal parameters

An ethogram was created to monitor and record gestational and perinatal parameters, in which the researchers took detailed notes of the events observed and their durations (in minutes), Throughout the observation process, the researchers remained outside the maternity bay to make sure human presence would not directly impact maternal behavior nor the physiological outcomes related to foaling and newborn adaptation.

**Fig. 1:**
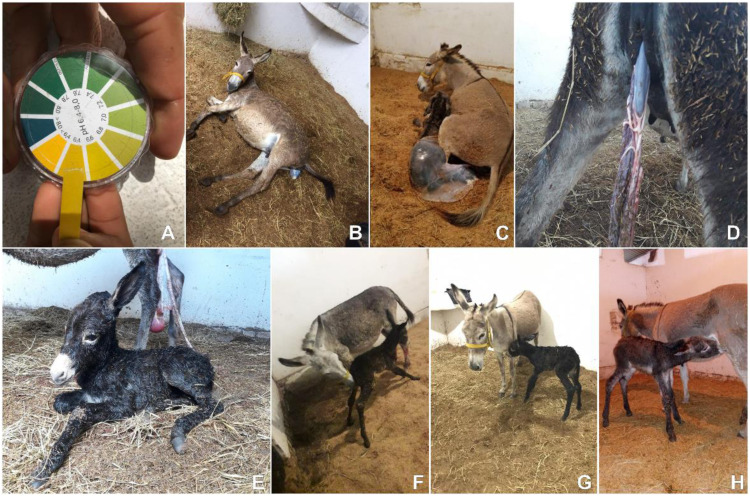
A) pH strip; B) Delivery; C) Placenta break; D) Placenta expulsion; E) Sternal recumbency; F and G) standing; H) First suckling.

The maternal and neonatal parameters assessed during parturition and immediate postpartum period included (Fig. 1):

• Foaling duration – period between the placental bag showing until the full expulsion of the foal;
• Time to placental expulsion – period between the placental sac showing and the full expulsion of placental annexes;
• Foal in sternal recumbency – period between foal expulsion until achieving sternal recumbency;
• Suckling reflex – assessed through suckling movement with the lips looking for teats;
• Standing – period between foal expulsion until achieving a standing position;
• Time to colostrum intake – period between foal expulsion and the first suckling event;
• Meconium release – period between foal expulsion and release of first feces;
• Urine release – period between foal expulsion and first urination;
• Newborn weight – determined in the first 24 h of life using a 200 kg digital scale (WelmyⓇ) with 50 g precision and a helper;
• Placenta weight – after the placenta was rinsed with water, it was weighed in a digital scale in a plastic container;
• Jenny weight – measured with a digital scale (FilizolaⓇ) with 500 g precision in the first 24 h after foaling.

### 2.9 Statistical analyses

The data on jennies and foals in the perinatal period were submitted to Kolmogorov-Smirnov normality test followed by analysis of variance using the statistical software GraphPad InStat (version 3.06). In addition, the values of mammary secretion pH monitored from the 11th month of gestation onwards and the number of days before foaling were submitted to Pearson correlation analysis. All analyses adopted significance level of 5%.

## 3 Results

**Fig. 2:**
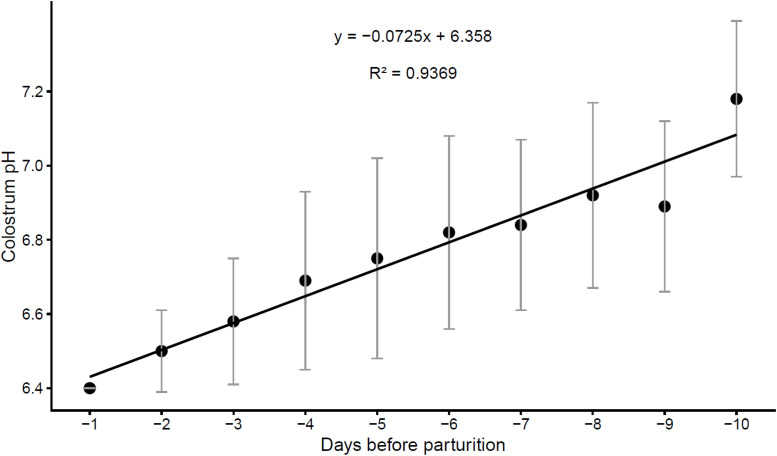
Mean mammary secretion pH curve of jennies of the Nordestino ecotype over the ten days before delivery.

No difference (p > 0.05) was observed between the experimental groups for mammary secretion pH in the ten days prior to parturition. A strong correlation was seen in both groups between the days before foaling and mammary secretion pH (Fig. 2). Ten days before parturition, average pH was 7.18 and, as foaling neared, pH progressively decreased down to 6.4. Parturition took place, on average, 39.14 ± 29.62 h after pH reached that value.

Foaling took place with no intercurrences and required no human intervention in all cases. No differences (p > 0.05) were observed between primiparous and multiparous jennies for weight, gestation length, foaling duration, time to release the placenta, placenta weight, placenta weight/mother weight ratio, or placenta weight/newborn weight ratio (Table 2). A difference (p < 0.05) was seen between the experimental groups only concerning newborn weight, with foals from multiparous jennies being heavier (18.92 ± 1.99 kg) than those from primiparous mothers (16.35 ± 2.27 kg).

**Table 2:** Gestational parameters of primiparous and multiparous jennies of the Nordestino ecotype.

	Primiparous	Multiparous	p-value
Jenny weight (kg)	178.98 ± 16.37	187.20 ± 15.04	0.1959
Gestation length (days)	367.00 ± 12.23	365.90 ± 9.59	0.8508
Foaling duration (min)	21.25 ± 6.85	20.9 ± 5.84	0.9194
Placenta release (min)	21.25 ± 16.46	42.6 ± 60.68	0.4592
Placenta weight (kg)	2.31 ± 0.27	2.28 ± 0.34	0.8360
Placenta/mother weight (%)	1.12 ± 0.15	1.31 ± 0.29	0.1859
Newborn weight (kg)	16.35b ± 2.27	18.92a ± 1.99	0.0047
Newborn/mother weight (%)	9.53 ± 0.85	10.50 ± 0.93	0.0770
Placenta/newborn weight (%)	11.78 ± 0.88	12.48 ± 2.40	0.5422

Different letters indicate difference according to F-test (p < 0.05).

Regarding the behavioral parameters of newborns, no difference (p > 0.05) was seen between the experimental groups for time to sternal recumbency, suckling reflex, meconium release, or first urination. On the other hand, the newborns from multiparous jennies were quicker to stand and for first suckling (Table 3).

**Table 3:** Behavioral parameters, in minutes, of newborns from primiparous and multiparous jennies of the Nordestino ecotype.

	Primiparous	Multiparous	p-value
Sternal recumbency	9.25 ± 13.85	3.55 ± 5.57	0.2413
Standing	46.25a ± 29.80	22.90b ± 11.63	0.0285
Suckling reflex	42.25 ± 41.48	24.5 ± 7.78	0.1459
First suckling	93.50a ± 28.20	57.00b ± 21.62	0.0111
Meconium release	134.25 ± 45.47	110.30 ± 55.67	0.4087
First urination	743.50 ± 530.57	683.10 ± 238.26	0.7382

Different letters indicate difference according to F-test (p < 0.05).

## 4 Discussion

In equines, the reduction in mammary secretion pH is an important factor to predict foaling. Colorimetric strips are widely used for that monitoring as it is a simple, convenient, and low-cost method that only requires commercially-available strips and a drop of mammary secretion.

According to Canisso and Ellerbrock (2016), the normal mammary secretion pH before foaling in equines ranges from 8.0 to 8.5 and gradually decreases to 6.4, which is corroborated in the present study (pH 6.4 ± 0.08), by Korosue et al. (2013) in mares (pH 6.4 ± 0.1), and by Magalhaes et al. (2021) (pH 6.6 ± 0.02), who observed a decrease in mammary gland secretion in multiparous jennies of the Dezhou breed.

During pre-colostrum production, mammary secretion pH gradually decreases, which is associated with the action of the carbonic anhydrase enzyme. That enzyme rapidly catalyzes the conversion of carbon dioxide (CO2) into carbonic acid (H2CO3), which dissociates into hydrogen ions (H+) and bicarbonate (HCO3-). The increase in H+ in the medium results in the acidification of the secretion, i.e., a reduction in mammary secretion pH. That initial acidification may have a protective role by hindering the growth of pathogenic microorganisms and favoring the stability of important bioactive compounds such as immunoglobulins. Therefore, the activity of carbonic anhydrase in the mammary gland cells directly contributes to physicochemical changes in colostrum soon after parturition (Canisso et al., 2013).

The similar mean body weight between the groups of primiparous and multiparous jennies, at an average of 183.09 ± 15.3 kg, can be attributed to the uniformity of the biotype imposed by the natural selection process.

Although the gestation length in equine females was influenced by parity, environmental factors, and foal sex in prior studies (Silva et al., 2021; Talluri et al., 2016), the gestation length of multiparous and primiparous jennies in the present study was similar at 365.90 ± 9.59 and 367 ± 12.23 days, respectively. Compared with females of other domestic mammals, equine females usually have longer gestational periods, ranging between 371 and 400 days in jennies (Carluccio et al., 2015). Magalhaes et al. (2021) reported an average gestational length of 374 ± 8.5 days in jennies of the Dezhou breed. Those authors also investigated the influence of newborn sex on the gestational period, however, they found no difference between gestations that resulted in male or female foals.

Studies on bovines showed that paternal genotype may influence gestation length since genetic characteristics inherited from the male affect embryo development and fetus growth and impact duration to foaling; studies on Holstein bovines identified paternal genes associated with that variation (Fang et al., 2019). Although the present study did not investigate such effect, it represents a plausible hypothesis to explain the lack of difference between primiparae and multiparae given that all jennies mated with the same donkey.

The start of foaling is related to a complex hormonal cascade, in which fetal cortisol plays a key role. That hormone is responsible for promoting not only the maturation of fetal organs, but also for preparing the fetus to the extrauterine medium (Nagel & Aurichb, 2022). In the present study, parturition of jennies of the Nordestino ecotype took 21.08 min on average, which is shorter than the range reported by Silva and Oliveira (2015) for equine females of 30 min to 4 h. Nevertheless, that time was longer than the one observed by Mazzatenta et al. (2019), who reported an average of 18.3 min for fetal expulsion in jennies of the Martina Franca breed.

In equines, the release of fetal annexes of the newborn takes place, physiologically, between 15 min and 3 h after foaling (Silva & Oliveira, 2015). In the present study, the multiparous jennies took 42.06 min on average for placental expulsion, which is close to the 40.4 min reported by Mazzatenta et al. (2019) for multiparous jennies of the Martina Franca breed.

The lack of differences in placenta weight and placental indices (placenta/mother weight and placenta/newborn weight) suggest that the similar body build and weight of the jennies in the present study may have contributed to uniform placental development. In the study by Nagel et al. (2020), the relative weight of the placenta did not differ between equines of small (Shetland) and medium (Haflinger) sizes, but was higher in large-size (Warmblood) horses. Moreover, foal weight at parturition in relation to placenta weight in that study was higher in foals of the large-size breed and did not differ between foals from mares of small and medium size.

The placental characteristics observed in Nordestino jennies were lower than those reported for horses (placenta weight of 5.7 kg), but similar to those described for ponies (placenta weight of 2.38 kg) (Mendoza & Toribio, 2024). In the present study, placenta weight corresponded to about 12% of foal weight at parturition, which is close to the 11% reported for horses by Mendoza and Toribio (2024). Wilsher and Allen (2003) point out that foal weight at parturition depends on the balance between mother-fetus contact and placental efficiency.

According to Klewitz et al. (2015), equine newborns should have at least 10% of the maternal weight at parturition since lower values may be associated with higher risk of neonatal diseases and possible reproductive compromises in the future. In the present study, the mean weight of foals from primiparous jennies was 9.5% maternal weight (Table 2). In contrast, at parturition, multiparous jennies produced foals weighing 10.5% of maternal weight on average. Those data suggest the greater placental efficiency observed in multiparous jennies may contribute, at least partially, to the higher neonatal weights found for that group when compared with foals from primiparous mothers.

Studies have shown the effect of parity to be intrinsically related to intrauterine development of the foal, having a direct influence on morphophysiological aspects of the gestation. According to Wilsher and Allen (2003), parity acts as a modulating factor of the structural and functional characteristics of the placenta, impacting parameters such as vascularization, thickness of chorionic microvilli, and the efficiency of nutrient and oxygen transfer between mother and fetus. Those placental alterations play a key role in the optimization of the uterine environment, favoring the nutritional supply, oxygenation, and hormonal equilibrium required for adequate fetal growth and development.

The mean weights of newborns of the Nordestino ecotype (16.35 ± 2.27 kg for primiparous and 18.92 ± 1.99 kg for multiparous mothers) are lower than the 30.3 kg reported by Mazzatenta et al. (2019) for donkey foals of the Martina Franca breed. Such discrepancy is plausibly explained by the phenotypical and zootechnical differences between the populations studied since Martina Franca is a large-size breed, with mean body weight around 360 kg, greater height, and larger bone and muscle development. In contrast, jennies of the Nordestino ecotype have smaller size, with mean body weight of 173.3 kg (Pimentel et al., 2014), adapted to semi-arid climate conditions, which translates into lower body weight and, consequently, lower newborn weight.

Foals of the Nordestino ecotype were quicker to stand up than those of the Martina Franca breed (62.3 min). The smaller size of the Nordestino ecotype, with shorter limbs, likely justifies that difference. Those values differed within the ecotype due to parity. The foals born from multiparous jennies took, on average, 22.90 ± 11.63 min to stand up, while those from primiparous mothers took significantly longer at 46.25 ± 29.80 min. That delay may be related to the lower weight and vigor of foals from primiparous mothers. That reflects what has been reported for equine foals, in which lighter newborns show greater initial motor difficulty and delayed first suckling.

The greater agility observed in foals from multiparous jennies may be related to the greater placental efficiency of those mothers, also reflected in greater weight at parturition. However, since the placental surface was not assessed, that hypothesis could not be verified. Moreover, the maternal experience of multiparous mothers in expressing care behaviors, such as self-cleaning and tactile, olfactive, and auditive stimuli, towards the newborn may have contributed to anticipating the standing reflex, thus favoring a quicker motor response in foals. That aspect is particularly relevant since delayed first suckling compromises the adequate absorption of immunoglobulins, which raises the risk of failure to transfer passive immunity.

Colostrum and passive immunity transfer have already been assessed in the Nordestino ecotype. Felix et al. (2022) observed that newborn foals of the Nordestino ecotype were agammaglobulinemic and, after colostrum intake, exhibited a rapid increase in circulating immunoglobulin G (IgG) concentration, reaching mean level of 1.64 g/dL at 6 h of life, with a peak of 3.07 g/dL at 17.9 h followed by a slight decrease at 24 h.

According to the literature, equine colostrum is considered as great quality when IgG concentration is above 8.0 g/dL (Knottenbelt et al., 2004). The colostrum of jennies of the Nordestino ecotype showed mean IgG concentration of 8.71 g/dL at time zero, which characterizes it as adequate for passive immunization of the foal (Felix et al., 2022). Those authors observed, however, that the IgG concentration in colostrum decreased by 93.5% within the first 24 h after foaling, reaching 0.56 g/dL, which shows the critical importance of early intake of colostrum for efficient absorption of immunoglobulins and establishment of passive immunity.

Since the foals from multiparous jennies were quicker to stand up, it is natural that they also showed greater agility for the first suckling. Given they stood up quicker than foals of the Martina Franca breed, it was also expected that foals of the Nordestino ecotype would be quicker for the first suckling. Mazzatenta et al. (2019) observed mean time of 87.1 min to first suckling in Martina Franca foals. Once again, that difference shows that, although belonging to the same species, donkeys of the Nordestino ecotype have distinct reproductive and adaptative particularities, possibly influenced by genetic, environmental, and physiological factors. That selective pressure favored more agile and responsive individuals, since rapid locomotion and early first suckling pose crucial adaptative advantages for survival in environments marked by more intense challenges.

Additionally, based on the data presented in Table 3, it was possible to estimate the interval between the moment foals achieved standing and the first suckling event. Foals from primiparous jennies took, on average, approximately 47.2 min from standing to first suckling, whereas foals from multiparous jennies showed a shorter interval, around 34.1 min. This pattern suggests not only greater motor agility but also potentially higher efficiency in the mare–foal interaction in multiparous jennies, either due to foal ability or the greater maternal experience in guiding the offspring to the udder.

Although other parameters such as time for sternal recumbency, suckling reflex, meconium release, and first urination did not significantly differ between groups, newborns from primiparous jennies were found to tend to be slower. Therefore, the results of the present study show that primiparity in donkeys of the Nordestino ecotype mainly impacts weight at parturition and neonatal vitality, with no significant impact on gestational physiology or placental indices.

Although the present study had a more direct approach towards the effect of parity, it is worth pointing out that maternal age was an equally relevant factor in the evaluation of gestational and perinatal parameters. In the case of the multiparous jennies included in this trial, the exact age of some of the animals could not be precisely determined since some were incorporated into the herd as adults. Nevertheless, the literature shows that age and parity often have concomitant effects on the intrauterine environment and fetal development. Studies on equines have shown that both age and parity influence aspects such as uterine blood flow, placental development, and weight at birth (Klewitz et al., 2015; Wilsher & Allen, 2003). In that context, it can plausibly be assumed that part of the differences attributed to parity may also reflect, albeit indirectly, effects associated with the physiological maturity of the jennies. Therefore, the influence of maternal age must be a relevant aspect to be explored in future investigations with greater control over that variable.

## 5 Conclusion

It is concluded that jennies of the Nordestino ecotype have reproductive and neonatal adaptations relevant to semi-arid conditions and stand out for their physiological and behavioral efficiencies that favor survival in that environment. The reduction in mammary secretion pH proved a helpful tool to predict foaling.

Moreover, foals from multiparous jennies manifest neonatal behaviors earlier. From a practical standpoint, that result shows the need for greater attention towards the monitoring of primiparous jennies since the longer interval between parturition and first suckling may increase the risk of failure in passive immunity transfer.

Those findings support the relevance of the rusticity of the Nordestino ecotype and of maternal experience as key factors for neonatal viability and sustainable management of the species.

## Funding

This work was supported by the Universidade Federal Rural de Pernambuco (UFRPE); Programa de Pós-Graduação em Ciência Animal e Pastagens (PPGCAP) of Federal University of Pernambuco Agreste (UFAPE) and the Coordenação de Aperfeiçoamento de Pessoal de Nível Superior (CAPES).

## CRediT authorship contribution statement

**I.M. de Farias:** Writing – review & editing, Writing – original draft, Investigation, Data curation. **J.M. Santiago:** Writing – review & editing, Writing – original draft, Project administration, Methodology, Conceptualization. **G.G. de Sobral:** Writing – review & editing, Writing – original draft. **A.H. da Silva:** Writing – review & editing, Writing – original draft, Investigation, Formal analysis, Data curation. **N.A. Felix:** Investigation. **D.S.A. Pinto:** Investigation. **E.F. de Melo Júnior:** Investigation. **K.M.L. da Silva:** Investigation. **G.F. Carneiro:** Writing – review & editing, Writing – original draft. **V.N. Maia:** Investigation. **A.P.G. Pinto:** Formal analysis, Validation, Visualization, Writing – review & editing. **L.L. da Rocha:** Formal analysis, Methodology, Validation, Writing – review & editing. **I.V.F. Gonzaga:** Investigation, Methodology, Validation, Writing – review & editing. **F.M.P. Taran:** Investigation, Validation, Writing – review & editing. **J.E.C. Lucena:** Writing – review & editing, Writing – original draft, Resources, Project administration, Methodology, Funding acquisition, Conceptualization.

## Declaration of competing interest

The authors declare the following financial interests/personal relationships which may be considered as potential competing interests:

Juliano Martins Santiago reports financial support was provided by Coordenação de Aperfeiçoamento de Pessoal de Nível Superior. If there are other authors, they declare that they have no known competing financial interests or personal relationships that could have appeared to influence the work reported in this paper.

## Data Availability

The data that support the findings of this study are available from the corresponding author upon reasonable request.

## References

[bib0001] Abraham M., Bauquier J. (2021). Causes of equine perinatal mortality. The Veterinary Journal.

[bib0002] Canisso I., Ellerbrock R. (2016). Proceedings of the 62nd Annual Convention of the American Association of Equine Practitioners (AAEP).

[bib0003] Canisso I.F., Ball B.A., Troedsson M.H., Silva E.S.M., Davolli G.M. (2013). Decreasing pH of mammary gland secretions is associated with parturition and is correlated with electrolyte concentrations in prefoaling mares. Veterinary Record.

[bib0004] Carluccio A., Gloria A., Veronesi M.C., De Amicis I., Noto F., Contri A. (2015). Factors affecting pregnancy length and phases of parturition in Martina Franca jennies. Theriogenology.

[bib0005] Carroll C.L., Huntington P.J. (1988). Body condition scoring and weight estimation of horses. Equine Veterinary Journal.

[bib0006] Fang L., Jiang J., Li B., Zhou Y., Freebern E., VanRaden P.M., Cole J.B., Liu G.E., Ma L. (2019). Genetic and epigenetic architecture of paternal origin contribute to gestation length in cattle. Communications Biology.

[bib0007] Felix N.A., Lucena J.E.C., Santiago J.M., Silva K.M.L., Melo Júnior E.F., Pinto D.S.A., Silva A.R., Sobral G.G. (2022). Evaluation of colostrum quality and passive immunity transfer in donkeys of the Brazilian Nordestino ecotype via different methods. Emerging Animal Species.

[bib0008] Holyoak, R., & Freeman, D.W. (2003). Foaling management and care of the nursing foal. Oklahoma Cooperative Extension Service, ANSI-3985.

[bib0009] Klewitz J., Struebing C., Rohn K., Goergens A., Martinsson G., Orgies F., Probst J., Hollinshead F., Bollwein H., Sieme H. (2015). Effects of age, parity, and pregnancy abnormalities on foal birth weight and uterine blood flow in the mare. Theriogenology.

[bib0010] Knottenbelt D.C., Holdstock N., Madigan J.E. (2004).

[bib0011] Korosue K., Murase H., Sato F., Ishimaru M., Kotoyori Y., Tsujimura K., Nambo Y. (2013). Comparison of pH and refractometry index with calcium concentrations in preparturient mammary gland secretions of mares. Journal of the American Veterinary Medical Association.

[bib0012] Magalhães H.B., Canuto L.E.F., Canisso I.F. (2021). Electrolytes and pH of mammary gland secretions assessments to detect impending parturition and associations with placental and neonate features in donkeys. Journal of Equine Veterinary Science.

[bib0013] Mazzatenta A., Veronesi M.C., Vignola G., Ponzio P., Carluccio A., De Amicis I. (2019). Behavior of Martina Franca donkey breed jenny-and-foal dyad in the neonatal period. Journal of Veterinary Behavior.

[bib0014] Mendoza F., Toribio R.E., Wong D.M., Wilkins P.A. (2024). Equine neonatal medicine.

[bib0015] Messias T., Moura E.O., Sant’Ana A.M.S., Lucena J.E.C., Pacheco M.T.B., Queiroga R.C.R.E., Weiblen R., Palo P. (2021). Challenges and perspectives for exploiting donkey milk in the Brazilian Northeast. Ciência Rural.

[bib0016] Nagel C., Aurich C. (2022). Induction of parturition in horses: From physiological pathways to clinical applications. Domestic Animal Endocrinology.

[bib0017] Nagel C., Melchert M., Aurich C., Aurich J. (2020). Differences in endocrine and cardiac changes in mares and her fetus before, during, and after parturition in horses of different size. Animals.

[bib0018] National Research Council (2007).

[bib0019] Pimentel M.M.L., Pinheiro M., Maia Filho H., Sakamoto S.M., Nobre F.V., Dias R.V.C. (2014). Parâmetros biométricos de asininos (Equus asinus) utilizados em provas de corrida no estado do Rio Grande do Norte. Acta Veterinaria Brasilica.

[bib0020] Ribeiro N.L., Medeiros G.R., Gomes I.L.C., Nascimento G.V., Santos S.G.C.G. (2023).

[bib0021] Silva F.L., Oliveira F.C. (2015). Parto e cuidados neonatais em equinos. Revista Brasileira de Reprodução Animal.

[bib0022] Silva G.C., Nogueira C.E.W., Mazzo H.C., Dallmann P.B.J., Silva R.B., Curcio B.R. (2021). Factors affecting gestation length in mares: Literature review. Research, Society and Development.

[bib0023] Talluri T.R., Arangasamy A., Singh J., Ravi S.K., Pal Y., Legha R.A., Raj M.A., Chopra A., Singh R.K., Tripathi B.N. (2016). Factors affecting length of gestation in artificially inseminated Marwari mares of India. Asian Pacific Journal of Reproduction.

[bib0024] Wilsher S., Allen W.R. (2003). The effects of maternal age and parity on placental and fetal development in the mare. Equine Veterinary Journal.

